# Wave generation and transmission in multi-scale complex media and structured metamaterials

**DOI:** 10.1098/rsta.2022.0141

**Published:** 2022-09-05

**Authors:** Alexander Movchan, Gennady Mishuris, Federico Sabina

**Affiliations:** ^1^ Department of Mathematical Sciences, University of Liverpool, Liverpool, UK; ^2^ Aberystwyth University, Institute of Mathematics and Physics, Aberystwyth, UK; ^3^ Universidad Nacional Autonoma de Mexico, Instituto de Investigaciones en Matematicas Aplicadas y en Sistemas, Mexico City, Mexico

Rapid technological developments in material science, including wave generation, control and detection, accompanied by new mathematical and computational approaches, are driven by the design of new generations of advanced smart dynamic materials used in a wide range of applications in engineering and physics.

In the beginning of this introduction, we would like to pay our tribute to Dr Rabindra Kumar Bhattacharyya who was a distinguished scholar and who started putting together the ideas for the volume on wave generation and transmission in real, elastic and smart media. Sadly, Dr Bhattacharyya became ill with COVID-19 and passed away in 2021. Dr Bhattacharyya had a vision connecting the fundamental science of wave theory with the applications in physics, engineering and materials science. As a brilliant applied mathematician, he developed special interests in mathematical models of elasticity, geophysics and wave propagation in complex media. Dr Bhattacharyya was very active in promoting science and art, and he was a Life Member of the Calcutta Mathematical Society and of the Indian Association for the Cultivation of Science. Dr Bhattacharyya has published extensively on the modelling of waves in micropolar thermoelastic media, Rayleigh waves in granular medium, random thermal granular media, wave propagation in random thermoelastic materials with memory, conducting magneto-viscoelastic solids, as well as computations of synthetic seismograms in real earth models, and mathematical models of geophysics. Dr Bhattacharyya was also known for his passion for art and poetry, as a member of the Bengal Literary Society at Calcutta (Bangiya Sahitya Parishad). He has given public addresses on the history of mathematics and published a substantial work on the poetry and songs of Rabindranath Tagore, including a significant monograph entitled ‘Rabindranath Tagore’s Poetry—A Scientific and Mathematical Interpretation’^1^. Dr Rabindra Kumar Bhattacharyya’s passion for mathematics, science, poetry and art was reflected in his wonderful teaching, which inspired many students and young scientists during his illustrious 60-year academic career.

Many of the ideas of Dr Bhattacharyya are embedded in the structure of the present theme issue, which reflects on an important area of knowledge and includes the recent results, both theoretical and experimental. Here, effort is made to incorporate research interlinking new mathematical methods and ideas, engineering studies, physics research and applications in the important area of wave generation and transmission in multi-scale complex media and structured metamaterials.

There are multi-scale structured materials capable of multi-functional behaviour according to pre-designed, sometimes unusual, dynamic anisotropy and inertial properties. The necessity and importance of the theoretical foundations combined with the ideas of practical implementation and engineering proof-of-concept applications are driven by attractive new concepts of wave dynamics in the context of wave control, wave generation and detection. In particular, dynamic defects in structured solids and elastic lattices, and chiral structured systems, which have a highly unusual dynamic response, will be discussed here in the context of Floquet–Bloch waves, as well as exponentially localized vibration modes. It is also noted that formally, equations, which describe elastic chiral systems, have a strong resemblance to formulations of quantum physics. Such an interlink appears to be useful both at the level of the fundamental theory and in applications.

The analytical and numerical approaches to the dynamic characterization of elastic waves in heterogeneous and anisotropic rocks and modelling of an earthquake, accompanied by a seismic assessment, are addressed in the theme issue and are timely and important to the current scientific strategies and interest in the research community. Reflecting on the relatively recent events related to vibrations and seismicity, examples include the L’Aquila earthquake (2009), the Ponte Morandi viaduct failure in Genoa (2018) and Blackpool earthquakes (2019). Although these events were predictable, with the technical data fully available to mathematical modellers and engineers, the guidelines existing at the time were affected by the gap between the mathematical modelling knowledge and the engineering standard practice related to the observations and routine technical maintenance of large-scale vibration systems. These examples emphasize the importance of novel approaches, which take into account the failure mechanisms in structured solids in the context of wave propagation and localization.

The theme issue brings together the fields of applied mathematics, solid mechanics, physics, structural and mechanical engineering and environmental science, with the main focus on the mathematics-engineering interface. The list of authors in the present theme issue includes leading experts in mathematical modelling of waves in structured media as well as internationally recognized physicists and engineers whose work contributes strongly to the practical impact and industrial applications. There is a good balance between contributions from senior researchers and young talented scientists, with the research of all the authors being on the leading edge of modern science. Modern analytical methods, new theoretical and numerical approaches and experimental novel studies, together with scientific reviews written by the internationally recognized experts, are reflected in the current theme issue.

## Data Availability

This article does not contain any additional data.

## Footnotes

1 Bhattacharyya RK. 2014 *Rabindranath Tagore's Poetry: A Scientific and Mathematical Interpretation*. Sarat Book House, India.

